# Indents

**Published:** 1917-06

**Authors:** 


### INDENTS

**☞ Brief Articles of Technical or Scientific Value will receive notice and comment. Contributions invited.**

*Rational Occlusion vs. Normal Occlusion*

The essential feature in the maintenance of any organ or tissue in the highest state of health and resistance and consequently of service, is the performance of function, but we must differentiate between activity and function on the one hand and between function and strain on the other. So-called normal occlusion, like perfect mouth sanitation, is a beautiful ideal which we must keep always before us as our standard of perfection, but it must be remembered that normal occlusion is but a part of a larger scheme in which normal conformation, normal arrangement, normal apposition, and normal stability and resistance to stress must all play their parts, and that these conditions are necessary to a successful restoration of occlusion—indeed, I may say, for its tolerance. Where these conditions are not fulfilled I do not believe normal occlusion can be established and maintained. Occlusal contact of such character and force as to produce a degree of stress which would constitute mere performance of function for a tooth of normal stability and resistance will produce strain in the case of one less stable and resistant. Thus that which constitutes normal occlusion in the one case may be traumatic occlusion in the other; while what would be considered an insufficient occlusal contact and impact in the first case would in the second case be rational, though not normal.

In the light of these facts, then, I feel justified in concluding that while normal occlusion is a fixed and absolute relation between the occluding teeth regardless of the circumstances and relations of stress and resistance in the parts, rational occlusion is a condition in which the extent or area and character of the surfaces, their occlusal relations, and the force and direction of the impact are not necessarily those absolute conditions and relations implied by normal occlusion, but rather an adjustment of all these conditions and relations to the degree of resistance afforded by the stability of anchorage of the pier teeth; in other words, a scheme in which the stability and security of the pier teeth is the fixed point and the basis of calculation and operation. This is what I have chosen to call rational occlusion *versus* normal occlusion. If it be heresy, I am a heretic. "Bear ye one another's burdens" is the great spiritual principle through the exercise of which those less fit are brought into serviceable relations with their fellows, and the standard of efficiency of society as a whole is thus placed upon a higher plane. It is no less applicable to the dental family than it is to society at large.

I dwell on these things because many years of experience have taught me that durability is measured largely by the degree in which stress is balanced or overbalanced by resistance, and that where normal resistance is not available, stress and consequently the conditions and relations which regulate stress, must be modified to meet the demands of this lessened resistance. I repeat, then, that it is the business and the duty of the dental engineer to determine and coordinate resistance and stress at every point, avoiding inactivity on the one hand and strain on the other, and thus as far as possible to foster function, and so increase the usefulness as well as lengthen the life of the restoration.—J. K. Burgess in May *Cosmos.*

*Amalgam Filling in the Lungs*

Miss C., age twenty-six, while under gas anesthesia, aspirated an amalgam filling into the left lung. The crown of the tooth, a lower molar, was a mere shell, and crushed under the grasp of the forceps. The dentist, a very skilful man, when he discovered that the large amalgam filling was missing, asked the patient to have a radiograph taken. As she had no distressing symptoms she thought this to be unnecessary. Severe coughing paroxysms and sweating supervened; there was neither hemoptysis nor expectoration.

Under local anesthesia I passed the bronchoscope through the mouth, and found a diffused left bronchitis. The filling was found tightly embedded in the swollen mucosa and partially hidden by an annular edema of the invaded bronchial wall. The filling was disimpacted with a pair of sidecurved bronchoscopic forceps and extracted. The patient left for home the next morning, and in a few days the cough had entirely disappeared. The patient has since remained perfectly well. The bronchoscopic removal, including the application of the local anesthetic, required seven minutes and fifty-four seconds.

A portion of a tooth, or even a whole one, requires an excellent radiograph to show it, if it contain no metallic filling. Fluoroscopy, if negative, is valueless. No dependence should be placed upon the absence of symptoms, because in most cases of foreign bodies, other than vegetable substances, there is a quiescent period during which there are no symptoms referable to the air passages, and even cough may be totally absent. While there is no need for desperate haste, yet as a rule, the sooner bronchoscopy is done the easier the removal will be.—C. Jackson, M.D., in May *Dental Cosmos.*

*Painless Devitalizing Paste* Take of arseneous acid, ground fine, one and one-quarter grams; powdered cocain hydrochlorate, two and one-half grams; campho-phenique liquid, enough to make a creamy paste. Apply on a small pledget of cotton in a dry cavity and seal for forty-eight hours, when the pulp will be dead.—B. L. Thorpe in *Review.*

*Ionic Medication, with Special Reference to the Treatment of Granuloma and Pyorrhea*

It is difficult to realize that this article and that of Prinz in the April *Cosmos* treat of the same subject. The rationale of this method is succinctly stated as follows: "The advantage obtained by ionic medication in the use of ions over the ordinary osmotic methods is that it is possible to concentrate and drive the medicine into the tissues where, in its passage, it acts on an area below the surface which is not reached when a wash is used to irrigate or bathe the surface."

Some of the principal and best known ions used therapeutically are, under the antiseptic group, zinc, copper, mercury, silver and iodin, ions, all effective and useful for the treatment of gingivitis, pyorrhea, chronic alveolar abscesses, granuloma, fistulous tracts, septic root canals, epulis, stomatitis, etc.; under the anesthetic group, cocain, novocain, and allied alkaloids, useful for hypersensitive dentin, pulp, gum tissue and mucosae—adrenalin may be added for hemostatic effect; under miscellaneous groups, argyrol, useful in stomatitis, salicylic and quinin ions, advocated for trigeminal and occipital neuralgia. The most striking effects of ionization are to be seen in the treatment of granuloma and pyorrhea, and consequently Sturridge outlines his form of treating these conditions.

He advises the use of a three per cent. solution of zinc chlorid ($ZnCl_2$) and a platinum or zinc point at the positive pole—that is, in the root canal. The zinc cations are repelled from this positive pole, and tend to migrate *through* the tissues to the cathode. It is in the initiation of this migration that their therapeutic action is accomplished. The strength of the current approximates roughly that advised by Prinz (about 5 M.A.). The length of application is not given, but "The granuloma is ionized two or three times at intervals of a few days," and then the root canal is filled by Rhein's method of protruding guttapercha points. Prinz ascribes the beneficial result of this method to the concentration of chlorin anions in the apical and periradicular regions.

Sturridge is satisfied that pyorrhea can be cured by ionic medication with methodical attention to many diagnostic details, which are just as important as the treatment. Ionization is adequate to deal with the bacterial phase of the disease. ''Perfect instrumentation'' is an absolute prerequisite. Then a small pear-shaped zinc anode, wrapped with a few shreds of cotton and saturated with a three per cent. solution of $ZnCl_2$ is passed into the pockets and interspaces of the affected teeth. The patient holds the cathode in his hand. The anode is held steadily at the bottom of the pocket in general for a minute or two (with about 5 M.A.), after which the electrode can be removed, but in perfect contact, to an adjoining pocket. The treatment is repeated three times a week at first, and twice a week later, until all septic symptoms disappear.

A clinical record of a great number of cases assures Sturridge that not only can very bad cases of pyorrhea be cured by the aid of ionization, but that from year to year there is to be noted a steady improvement in the general condition of the mouth, so that the teeth are more comfortable after ten years than the first year or two after the cure had been effected.—Ernest Sturridge. An extract from Journal of Allied Societies in May *Dental Cosmos.*

